# New relative intensity ambulatory accelerometer thresholds for elderly men and women: the Generation 100 study

**DOI:** 10.1186/s12877-015-0093-1

**Published:** 2015-08-04

**Authors:** Nina Zisko, Trude Carlsen, Øyvind Salvesen, Nils Petter Aspvik, Jan Erik Ingebrigtsen, Ulrik Wisløff, Dorthe Stensvold

**Affiliations:** K.G. Jebsen Center of Exercise in Medicine at Department of Circulation and Medical Imaging, Faculty of Medicine, Norwegian University of Science and Technology, Trondheim, Norway; Department of Cancer Research and Molecular Medicine, Faculty of Medicine, Norwegian University of Science and Technology, Trondheim, Norway; Liaison Committee between the Central Norway Regional Health Authority (RHA) and the Norwegian University of Science and Technology (NTNU), Trondheim, Norway; Institute for Science in Sport, Norwegian University of Science and Technology, Trondheim, Norway

**Keywords:** Cut-points, Accelerometer, Actigraph, Elderly, Physical activity, Relative intensity, VO_2max_

## Abstract

**Background:**

Public health initiatives world-wide recommend increasing physical activity (PA) to improve health. However, the dose and the intensity of PA producing the most benefit are still debated. Accurate assessment of PA is necessary in order to 1) investigate the dose–response relationship between PA and health, 2) shape the most beneficial public health initiatives and 3) test the effectiveness of such initiatives. Actigraph accelerometer is widely used to objectively assess PA, and the raw data is given in counts per unit time. Count-thresholds for low, moderate and vigorous PA are mostly based on absolute intensity. This leads to largely inadequate PA intensity assessment in a large proportion of the elderly, who due to their declining maximal oxygen uptake (VO_2max_) cannot reach the moderate/vigorous intensity as defined in absolute terms. To resolve this issue, here we report relative Actigraph intensity-thresholds for the elderly.

**Methods:**

Submaximal-oxygen-uptake, VO_2max_ and maximal heart rate (HR_max_) were measured in 111 70–77 year olds, while wearing an Actigraph-GT3X+. Relationship between VO_2max_ percentage (%), counts-per-minute (CPM) and gender (for both the vertical-axis (VA) and vector-magnitude (VM)) and VO_2max_% and HR_max_% was established using a mixed-regression-model. VM-and VA-models were tested against each other to see which model predicts intensity of PA better.

**Results:**

VO_2max_ and gender significantly affected number of CPM at different PA intensities (*p* < 0.05). Therefore, intensity-thresholds were created for both men and women of ranging VO_2max_ values (low, medium, high). VM-model was found to be a better predictor of PA-intensity than VA-model (*p* < 0.05). Established thresholds for moderate intensity (46−63 % of VO_2max_) ranged from 669–3367 and 834–4048 CPM and vigorous intensity (64−90 % of VO_2max_) from 1625–4868 and 2012-5423CPM, for women and men, respectively. Lastly, we used this evidence to derive a formula that predicts customized relative intensity of PA (either VO_2max_% or HR_max_%) using counts-per-minute values as input.

**Conclusion:**

Intensity-thresholds depend on VO_2max_, gender and Actigraph-axis. PA intensity-thresholds that take all these factors into account allow for more accurate relative intensity PA assessment in the elderly and will be useful in future PA research.

**Trial registration:**

(ClinicalTrials.gov Identifier: NCT02017847, registered 17. December 2013)

## Background

Evidence shows that physical activity (PA) is beneficial for health [1, 2]. For that reason adults around the world (including elderly) are recommended to accumulate at least 150 min · wk^−1^ of moderate intensity PA or at least 75 min · wk^−1^ of vigorous intensity PA, with intensity defined in both relative (as a percentage of one’s cardiorespiratory fitness (CRF) i.e. maximal oxygen-uptake (VO_2max_), or one’s maximal heart rate (HR_max_)) and absolute (in metabolic equivalents of task (METs)) terms [3]. However, despite the well-documented health benefits of PA, only 20 % of the United States and 23 % of the Norwegian population meet the current PA recommendation [4, 5, 2]. Thus, proper assessment of PA is necessary because it (1) allows researchers to investigate the dose–response relationship between PA and health outcomes, (2) can help shape public health initiatives and interventions and (3) can be used to test the effectiveness of the public health initiatives and interventions that aim to increase PA [6].

Due to bias and limitations, originating mainly from recall error, associated with self-reported PA assessment methods, accelerometers are increasingly used to describe PA of a population [7, 8, 6]. It has been shown that 47 to 51 % of adults aged 70 or older meet current PA recommendations when the data are self-reported [9, 10], while the prevalence for the same population is much lower, with only 6 to 10 % meeting PA recommendation with objectively measured PA using accelerometers [10]. Accelerometers provide an objective measure of PA that remove many of the biases associated with self-reported measures and are appropriate for use in elderly [6].

Actigraph is an accelerometer commonly used in research on PA [6]. The raw Actigraph data is converted to counts per minute (CPM), which reflect the acceleration and hence the intensity of PA. The higher the CPM, the higher the acceleration of movement measured [11]. Thresholds used for estimation of different intensities of PA are determined from validation studies and are predominantly defined in terms of absolute intensity such as METs [6]. However, elderly may not be able to reach what is widely accepted as, in terms of accelerometer thresholds, absolute vigorous intensity PA due to declining CRF [12]. In younger adults moderate intensity PA is typically defined to range from 3.0−6.0 METs, while vigorous intensity PA is defined to range between 6.0−9.0 METs [3]. In contrast, it is estimated that in those 65–79 years of age, moderate intensity PA that is perceived as “somewhat hard”, corresponds to 3.2−4.7 METs, while vigorous intensity PA, perceived as “hard”, corresponds to 4.8 to 6.7 METs [12]. This changes further for elderly aged 80 or over. For them, moderate intensity PA corresponds to 2.0−2.9 METs and vigorous intensity PA to 3.0−4.3 METs [12]. It is difficult for those of very low CRF to reach PA intensities commonly defined as moderate and/or vigorous. However, most large scale population studies that include elderly use one-size-fits-all, fixed thresholds based on absolute intensity for all adults regardless of age or CRF [13, 14]. As CRF declines with age and CRF differs between men and women, relative intensity of effort required to perform PA will be influenced by both age and gender, and accelerometer intensity thresholds should be able to reflect that [12, 15, 16].

Therefore, the aim of this study was to use different fitness levels to create new relative intensity thresholds for light, moderate and vigorous PA for elderly men and women.

## Methods

### Participants

**Fig. 1:**
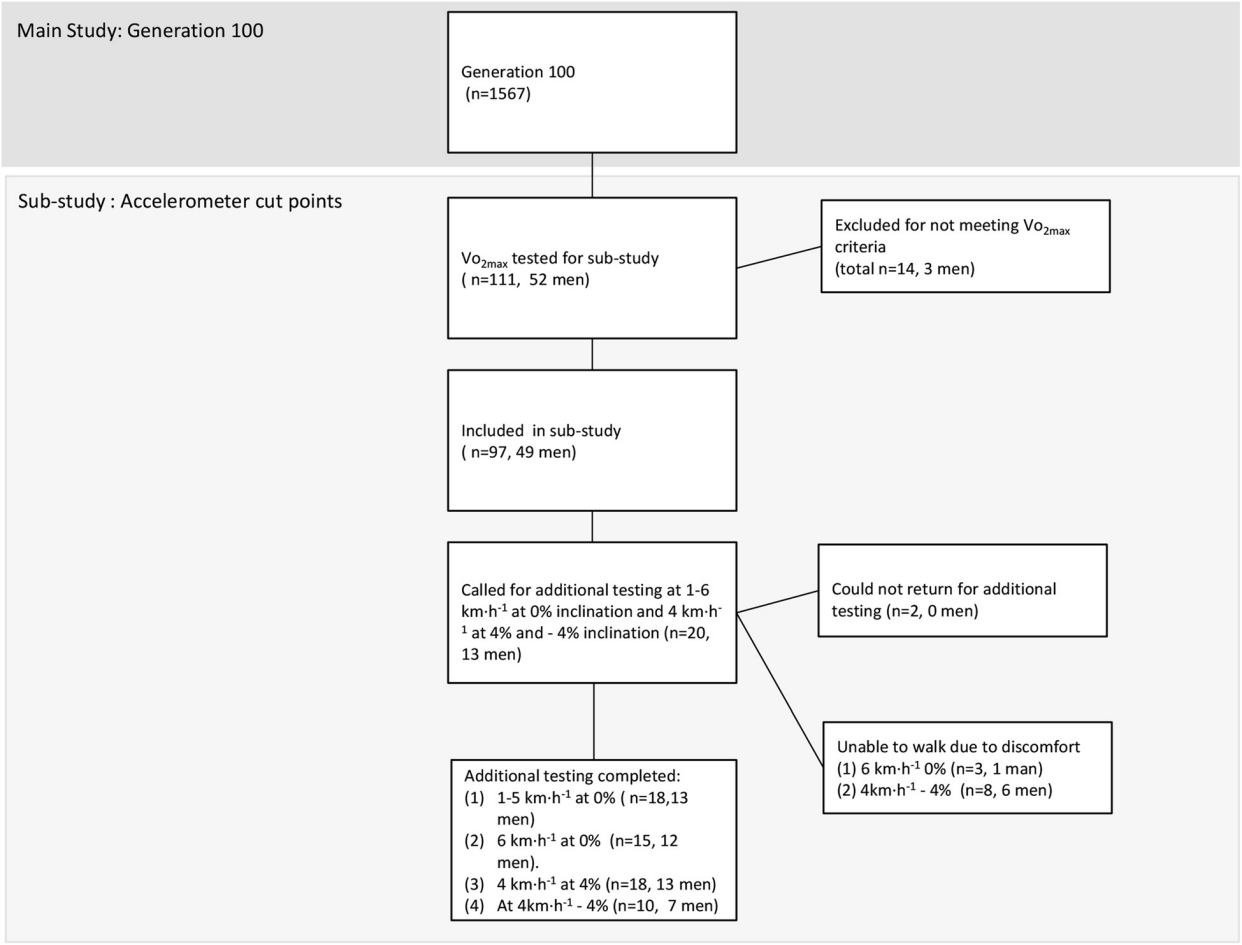
Study flow-chart

This study is a sub-study of Generation 100 (ntnu.edu/cerg/generation100) conducted in Trondheim, Norway. One hundred eleven participants (52 men), 70 to 77 years of age taking part in Generation 100 study, who came in for testing between Nov and Dec 2013, were asked to part-take in this sub-study. The protocol was approved by the Regional Committee for Medical Research Ethics and was registered in clinical trials (NCT02017847). Consent to participate in this sub-study was covered by the consent for the Generation 100 study, which was approved by the Regional Committee for Medical Research Ethics (2014/468). The inclusion and exclusion criteria for this sub-study were the same as for the Generation 100 study. Inclusion criteria: Born during 1936, 1937, 1938, 1939, 1940, 1941 or 1942, and able to complete the exercise programme (determined by the researchers). Exclusion criteria: Illness or disabilities that preclude exercise or hinder completion of the study, uncontrolled hypertension, symptomatic valvular, hypertrophic cardiomyopathy, unstable angina, primary pulmonary hypertension, heart failure or severe arrhythmia, diagnosed dementia, cancer that makes participation impossible or exercise contraindicated considered individually in consultation with physician, chronic communicable infectious diseases, test results indicating that study participation is unsafe, participation in other studies conflicting with participation in Generation 100. Flowchart of the current study is presented in Fig. 1.

### Accelerometer

Each participant wore an elastic waist belt with the Actigraph accelerometer unit (Manufacturing Technologies Inc. Health Systems, Model GT3X+, Shalimar, USA) placed over the right hip during all treadmill activity testing. Actigraph GT3X+ is a widely used accelerometer, and has been used in hundreds of large-scale studies including the NHANES, the European Youth Heart Study, and the Millennium Cohort Study [17–19]. The monitor measures acceleration in three different planes of motion: vertical (VA), antero-posterior and medio-lateral. All three planes combined yield vector magnitude (VM), a composite acceleration measure obtained using the following formula: $VM=\sqrt{{\left(\mathrm{V}\mathrm{A}\right)}^2+{\left(\mathrm{antero}-\mathrm{posterior}\right)}^2+{\left(\mathrm{medio}-\mathrm{lateral}\right)}^2\kern0.5em }$ [20].

Each monitor was initialized and synchronized with a digital clock, so that time could be synchronized with the ergospirometry system (MetaMax II, CORTEX Biophysik GmbH, Leipzig, Germany) and used while simultaneously measuring oxygen consumption (VO_2_) and accelerometry counts. Activity counts were stored in 10-s epochs and normal filter was used. After each individual walking/running treadmill test, Actigraph data was downloaded to a personal computer using Actilife 6.2.2 software (Manufacturing Technologies Inc. Health Systems, Model GT3X+, Shalimar, USA).

### Cardiopulmonary exercise testing

Prior to the cardiopulmonary exercise test (CPET) the following measurements were taken: body weight (measured to the nearest 0.1 kg using the bioelectrical impedance scale Inbody 720, BIOSPACE, Seoul, Korea), and height (measured to the nearest millimeter with a mechanical telescopic measuring stadiometer with large measuring range Seca 222, Hamburg, Germany).

CPET was performed at the core facility NeXt Move (www.ntnu.edu/dmf/nextmove) at the Norwegian University of Science and Technology (NTNU), Trondheim, Norway. VO_2_, heart rate (HR) and Actigraph data were collected simultaneously during walking/running on a treadmill at different workloads. All of our study participants completed a submaximal and VO_2max_ test. Of the 111 participants tested, 97 (49 men) met the Generation 100 VO_2max_ criteria and were included in the future analysis [21]. Participants were categorized into 3 different fitness categories (tertiles); low, medium and high. These fitness tertiles were derived from the Generation 100 population of 1567, whose VO_2max_ was measured as part of that study using CPET [21]. The cut-off values for low, medium and high fitness tertiles were <23.6, 23.6−29.8, >29.8 ml · kg^−1^ · min^−1^ for women and <27.0, 27.0−35.6, >35.6 ml · kg^−1^ · min^−1^ for men. All analyses were done separately for men and women.

VO_2_ was measured during different workloads on a treadmill using portable indirect calorimeter system (MetaMax II, Leipzig, Germany). Analysis of data was done in Metasoft version 3.9 (CORTEX Biophysik GmbH, Leipzig, Germany). HR was measured using a heart rate monitor (S610i, Polar Electro Oy, Kempele, Finland). Volume calibration was performed between every test, while gas calibration was performed after every 4^th^ test, or if ambient air measurements were rejected. Barometric pressure was calibrated daily before the startup of testing. System was calibrated against ambient air and a gas (Scott Medical Products, Breda, Netherlands) containing 5 % carbon dioxide and 15 % oxygen.

CPET was performed on a motorized treadmill (PPS55 Med, Woodway GmbH, Weil am Rhein, Germany). Participants had a treadmill familiarization period of 8–10 min during the warm-up. The warm-up workload was selected on the basis of perceived physical exertion level (Borg scale between 10 and 11) [22]. Participants were encouraged not to hold on to the railing of the treadmill during the testing. In case of loss of balance, they were asked to gently place their hand on the treadmill railing without exerting too much pressure. Participants wore an appropriately sized face-mask (Hans Rudolph, Germany) linked to the MetaMax II.

After the warm-up, an individualized protocol was used to measure VO_2max_. HR, VO_2_, ventilation (VE), inclination, speed, respiratory exchange ratio (RER) and perceived exertion (Borg scale) were recorded at two submaximal levels, Step 1 and Step 2, and at maximal effort. Step 1 was initiated from the inclination and speed derived from the warm-up period, and was a steady state measurement that lasted 3 min. For Step 2, inclination was increased by 2 % and lasted 2 min. After completion of steps 1 and 2, load was gradually increased about every minute until exhaustion or until VO_2max_ was reached. HR_max_ was calculated by adding 5 beats to highest observed HR during the test, as previously suggested [23]. Eighteen participants (13 men) had their VO_2_ assessed while walking 5 min on each 1, 2, 3, 4, 5 and 6 km · h^−1^ at 0 % inclination and on 4 km · h^−1^ at 4 % inclination, for 6 min. Three of the 18 participants were unable to walk at 6 km · h^−1^ (feeling uncomfortable walking on the treadmill at that speed). Due to balance problems and joint discomfort, only 10 of the 18 participants (7 men) were able to walk at −4 % inclination and 4 km · h^−1^ for 6 min.

### Data analysis

The accelerometer data, downloaded using ActiLife, was exported into a Microsoft Excel file and was placed alongside the corresponding time-synchronized oxygen consumption data. The average CPM and VO_2_ of the last 30 s of each step 1 and step 2 were calculated. The first 2 min and the last 30 s of each walking activity were excluded from the analysis to eliminate the transitions between workloads and the remaining minutes were averaged to reflect mean CPM and VO_2_ at each stage. This mean VO2 was then used to calculate the relative intensities (VO2max%) at each submaximal stage and their associated CPM. Low, moderate and vigorous intensity PA were defined as per Garber et al. in terms of VO_2max_ percentage (VO_2max_%) [3]. Mixed regression model was used to generate intensity thresholds.

### Statistical analysis

All statistical analysis was done using R software (R Foundation for Statistical Computing, Vienna, Austria). The relationship between CPM and VO_2max_%, CRF and gender was established using a mixed regression model. In the regression model, VO_2max_% values were specified as binomially distributed given fixed and random effects. CPM, fitness level and gender were the fixed effects, while participant identification number was a random effect. A random intercept and slope (for the square root of CPM) were included in the model to account for apparent heteroscedasticity. For each combination of fitness level and gender, we established CPM-VO_2max_ % relationship and defined intensity thresholds as per Garber et al. [3]. Same statistical method was followed to establish the relationship between fitness level and gender, VO_2max_% and HR_max_ percentage (HR_max_ %). The procedure was done for both VA and VM-CPM. VM-CPM-model was tested against VA-CPM-model using Vuong-closeness-test to see which model predicts intensity of PA better.

## Results

Physical characteristics of study participants are presented in Table 1. Height, weight, waist circumference and BMI differed significantly between the two genders (*p* < 0.05). CPET data are presented in Table 2. There were significant differences between men and women in all CPET parameters except inclination (*p* < 0.05).

**Table 1:** Physical characteristics of study participants

	Women	Men
	(*N* = 48)	(*N* = 47)
Height (cm)	163.9 ± 5.7	176.2 ± 6.6
Weight (kg)	66.2 ± 8.9	81.0 ± 10.6
BMI (kg/m2)	24.6 ± 3.3	26.1 ± 2.9
Waist circumference (cm)	88.6 ± 10.1	96.2 ± 8.2
Cardiovascular disease (%)	15.2	14.6
Diabetes (%)	2.2	8.3
Cancer (%)	13.0	16.7
Hypertension (%)	28.3	29.2
Stroke (%)	13.0	12.8
Physical ailments (%)	11.6	4.3
Prescription medication (%)	73.2	78.3

Values are presented as mean ± standard deviation (SD)

Abbreviations: Cardiovascular disease, diabetes, cancer, hypertension, stroke, physical ailments, prescription medication = obtained from self-report questionnaires filled as part of Generation 100 study. Cardiovascular disease was defined as any of the following: Heart attack or angina pectoris or heart failure or atrial fibrillation or other heart condition. *BMI* body mass index

**Table 2:** CPET values during maximal exertion

Variable	N	Mean ± SD	
		Men	Women
VO_2max_ (ml · min^−1^ · kg^−1^)	97	31.0 ± 6.5	26.3 ± 5.1^a^
VO_2max_ (L · min^−1^)	97	2.480 ± 0.557	1.755 ± 0.262^a^
VE_max_ (L · min^−1^)	97	92.7 ± 18.7	61.2 ± 9.9^a^
RER_max_ (VCO_2max_/VO_2max_)	97	1.16 ± 0.7	1.12 ± 0.60^a^
HR_max_ (beats · min^−1^)	97	151 ± 13	157 ± 13^a^
Treadmill inclination at max (%)	97	13 ± 4	12 ± 3
Speed at max (km · h^−1^)	97	5.6 ± 0.9	5.2 ± 0.6^a^

Values are presented as mean ± standard deviation (SD)

Abbreviations: *VO*_*2max*_ maximal oxygen uptake, *VE*_*max*_ maximal pulmonary ventilation, *RER*_*max*_ maximal respiratory exchange ratio, *HR*_*max*_ Maximal heart rate

^a^statistically significant difference between sexes (*p* < 0.05)

### Thresholds for activities of different intensity

**Fig. 2:**
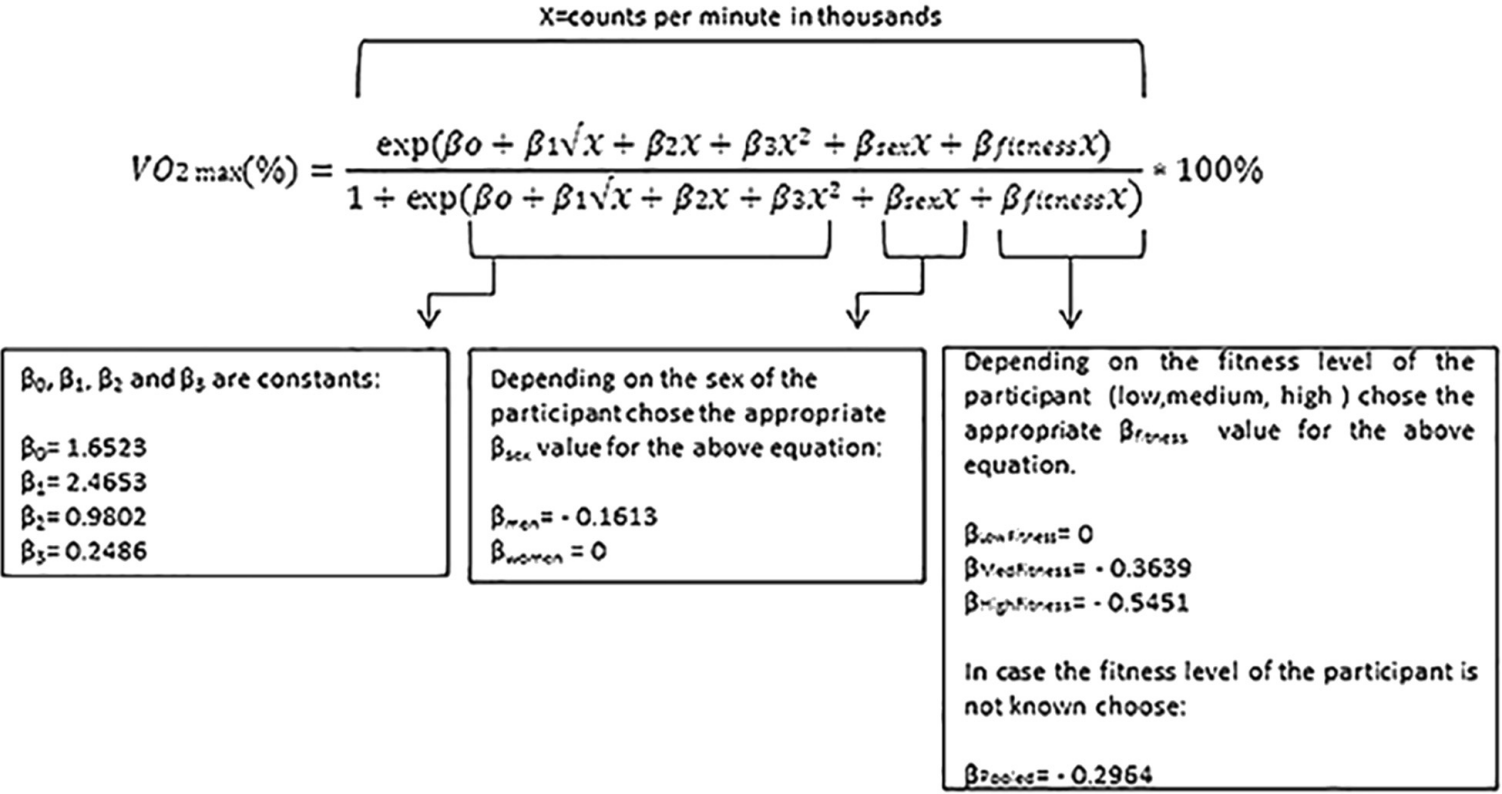
PA intensity (in VO2max%) prediction equation

**Fig. 3:**
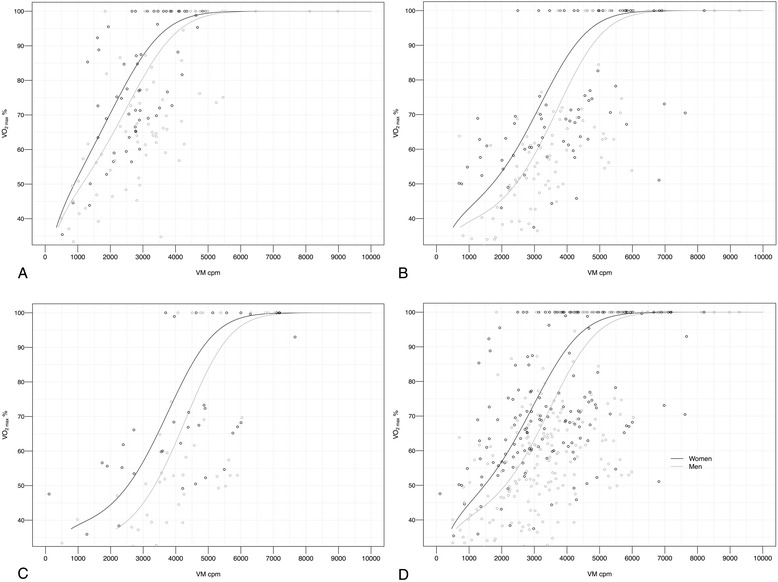
Intensity thresholds for men and women of (**a**) low fitness level, (**b**) medium fitness level, (**c**) high fitness level, (**d**) pooled fitness (all three levels of fitness pooled together). VO_2max_% = percentage of maximal oxygen uptake. Cpm = counts per minute. VM = vector magnitude

**Fig. 4:**
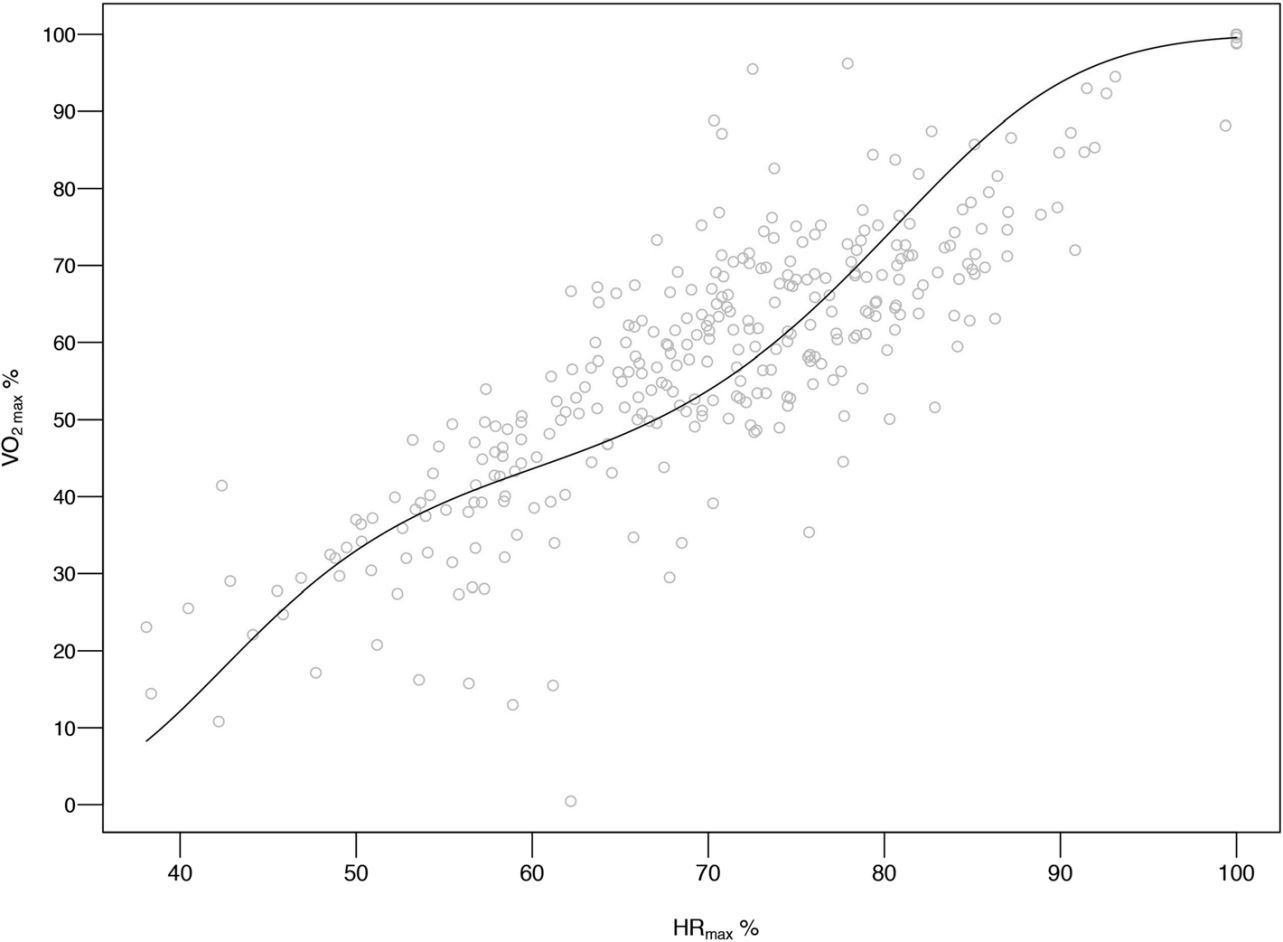
Relationship between percentage of maximal oxygen uptake and percentage of maximal heart rate. VO_2max_% = percentage of maximal oxygen uptake. HR_max_% = percentage of maximal heart rate

Both fitness level and gender were found to significantly affect CPM at different intensities (*p* < 0.001). Intensity thresholds were therefore determined separately for men and women of low, medium and high fitness tertiles (Table 3). In addition, pooled thresholds (with all fitness tertiles pooled together) were determined (Table 3). The VM-CPM model was significantly better than the VA-CPM in predicting intensity in terms of VO_2max_% (*p* < 0.05). Moderate intensity VM-CPM corresponding to 46−63 % of VO_2max_ differed for men and women and depending on fitness level of the individual ranged from 669–4048 CPM, while vigorous intensity VM-CPM corresponding to 64−90 % of VO_2max_ ranged from 1625–5423 CPM. Very light activity threshold was defined as CPM below the light intensity threshold (<37 % of VO_2max_). For prediction of PA intensity (VO_2max_%) based on CPM the equation presented in Fig. 2 was derived. Figure 3 shows the relationship between VO_2max_% and CPM for the VM-axis for low (A), medium (B) and high (C) fitness tertile as well as pooled (D) fitness tertiles. Furthermore, we established a relationship between VO_2max_% and HR_max_ % (Fig. 4).

**Table 3:** Actigraph thresholds for light to maximal intensity (in VO_2max_%) for men and women of different fitness tertiles

Relative Intensity			Vertical Axis (cpm)				Vector Magnitude (cpm)			
Intensity	VO_2max_ (%)		Pooled	Low Fitness	Medium Fitness	High Fitness	Pooled	Low Fitness	Medium Fitness	High Fitness
		Men	(*N* = 49)	(*N* = 17)	(*N* = 23)	(*N* = 9)	(*N* = 49)	(*N* = 17)	(*N* = 23)	(*N* = 9)
Light	37−45		56−266	51−204	57−289	60−421	611−1652	399−833	735−2004	2167−3047
Moderate	46−63		267−1971	205−1102	290−2254	422−3131	1653−3016	834−2011	2005−3285	3048−4048
Vigorous	64−90		1972−3878	1103−3001	2255−4097	3132−4747	3017−4581	2012−3688	3286−4802	4049−5423
Near-max to max	≥91		>3879	>3002	>4098	>4748	>4582	>3689	>4803	>5424
Intensity	VO_2max_ (%)		Pooled	Low Fitness	Medium Fitness	High Fitness	Pooled	Low Fitness	Medium Fitness	High Fitness
		Women	(*N* = 48)	(*N* = 16)	(*N* = 22)	(*N* = 10)	(*N* = 48)	(*N* = 16)	(*N* = 22)	(*N* = 10)
Light	37−45		60−212	55−175	62−223	67−269	465−1076	347−668	513−1268	790−2116
Moderate	46−63		213−1217	175−788	224−1380	270−2024	1077−2424	669−1624	1269−2662	2117−3367
Vigorous	64−90		1218−3157	789−2438	1381−3345	2025−3920	2425−4078	1625−3266	2663−4283	3368−4868
Near-max to max	≥91		>3158	>2439	>3346	>3921	>4079	>3267	>4284	>4869

Fitness tertiles for (1) women: Low <23.6 ml · kg^−1^ · min^−1^, Med = 23.6−29.8 ml · kg^−1^ · min^−1^, high > 29.8 ml · kg^−1^ · min^−1^,pooled = all tertiles of fitness together (2) men: Low < 27.0 ml · kg^−1^ · min^−1^, Med = 27.0−35.6 ml · kg^−1^ · min^−1^,High >35.6 ml · kg^−1^ · min^−1^, pooled = all tertiles of fitness together

Abbreviations: *VO*_*2max*_ Maximal Oxygen Uptake, *VA* vertical axis, *VM* vector magnitude, *cpm* CPM

## Discussion

In this study we established new Actigraph thresholds for very light, light, moderate and vigorous PA in an elderly population. This study is the first to use intensity thresholds based on fitness level and gender. We also show, for the first time, that the VM- CPM are a better predictor of relative intensity of PA compared to VA- CPM in this population, and their use should therefore be encouraged in future studies.

Relationship between accelerometer thresholds for different PA intensities and demographic characteristics (i.e. age and body weight) has been investigated by previous studies [24, 25]. These studies showed a clear difference between absolute intensity PA thresholds (in METs) created for the general population and absolute intensity PA thresholds (in METs) derived specifically for populations of different age groups or conditions (i.e. overweight, obese, type 2 diabetes) [24, 25]. However, even when absolute intensity PA thresholds are population specific, they may still underestimate or overestimate individual PA due to variation in CRF [26].

In a recent study by Miller et al. differences in absolute intensity PA thresholds were shown to vary with age at both moderate and vigorous intensity, with lower CPM observed in the older age groups [24]. Miller et al. ascribed this difference in thresholds between age groups to age-associated decline in CRF [24]. Our study results support the idea that those with lower CRF have lower CPM compared to those with higher CRF. However, what Miller et al. do not account for when using absolute intensity PA thresholds is the fact that some individuals remain fit well into advancing age, and may have CRF higher than those of younger age groups [26]. This may indicate that Miller et al. thresholds underestimate intensity of PA of the older subject with higher CRF while overestimating the intensity of the younger subject with lower CRF.

As previously suggested, absolute intensity PA thresholds are not appropriate for use in a population with ranging CRF levels regardless of age. This is because for those with lower CRF it is difficult to achieve absolute CPM commonly used to designate moderate or vigorous PA intensity (i.e. 3-6METs) [26]. Ozemek et al. demonstrated that CPM at moderate or vigorous PA intensity were significantly correlated to CRF (explaining 26 and 32 % of the variability, respectively) while only about 1 % of the variability in activity CPM could be explained by age and body mass index (BMI) [26]. They concluded that previously derived PA intensity thresholds based on age and BMI were unlikely to account for differences in CRF across age groups and body sizes, and that relative intensity PA thresholds, based on CRF, would be more appropriate than absolute intensity PA thresholds when classifying relative intensity of PA [26]. This is in agreement with the idea behind our study to create intensity PA thresholds that better reflect inter-individual variation in CRF.

Stevenson et al. study supports findings by Ozemek et al. They measured the time cardiac rehabilitation patients spent in different PA intensities. In this study they showed that when absolute intensity PA thresholds were applied, the patients did not spend any time in vigorous intensity PA, even though HR monitoring during the same time period indicated otherwise [27]. CRF for cardiac rehabilitation patients is very low (ranging from 14.5 ml∙kg^−1^∙min^−1^ for women to 19.3 ml∙kg^−1^∙min^−1^ for men) [28]. Similarly, some elderly have very low CRF, with some participants in our study having fitness level lower than the cardiac rehabilitation patients (lowest VO_2max_ measured in our study was 10.2 ml∙kg^−1^∙min^−1^ for women and 16.2 ml∙kg^−1^∙min^−1^ for men). As a result, these individuals with low CRF, cannot reach what is commonly defined as absolute moderate or vigorous PA intensity (3–6 METs) [12]. This has been empirically demonstrated in a study by Pruitt et al. that performed an evaluation of relative moderate and vigorous PA intensity thresholds in elderly. Ninety-three participants, 70–89 years old, were asked to walk 400 meters at their habitual pace while wearing Actigraph [29]. After 4 laps at this pace, they were asked to evaluate perceived intensity as ‘light’ or ‘hard’ and to slow down if they felt the pace was ‘somewhat hard’ and ‘very hard’. The thresholds that defined moderate to vigorous PA intensity ranged vastly, from as few as 149 CPM to as high as 3133 CPM. Pruitt et al. concluded that in older populations, PA thresholds should be more individualized to reflect variation in CRF. Our threshold-range for moderate and vigorous PA intensity is in line with findings by Pruitt et al. with moderate intensity and vigorous intensity thresholds ranging from 669 to 3048 and from 1625 to 5423 CPM, respectively, depending on CRF and gender.

The relative intensity PA thresholds in our study are based on CRF tertiles derived from a large population of 1567 individuals, whom we believe well represent the Norwegian elderly population. These tertiles, which differ between gendes, were then used to establish relative intensity PA thresholds for a population of relatively narrow age range (70–77 years). To our knowledge these are the first relative intensity PA thresholds that can be used on a large population of this age group. While Ozemek et al.’s individual relative intensity PA thresholds may not be appropriate for all settings and large surveillance studies, our relative intensity PA thresholds have a wider scope of use. Ozemek et al. PA thresholds require potential study participants to visit a laboratory and perform a treadmill protocol at different submaximal speeds while wearing an accelerometer and a HR monitor. This data would subsequently be used to create a relationship between CPM and participants’ HR, which in an appropriate regression equation would determine the activity count responding to moderate or vigorous intensity PA [26]. However, the advantage of our PA intensity thresholds is that they can be applied in studies where VO_2max_ testing is either not feasible or impossible (i.e. studies involving a very large number of participants). When the individual VO_2max_ is not known, pooled intensity PA thresholds (presented in Table 3) could be applied. Where VO_2max_ is known, gender and fitness level specific PA intensity thresholds (based on ACSM’s definition of intensity) presented in Table 3 could be applied, or equation presented in Fig. 2 could be used if one wants to define their own intensities in terms of VO_2max_ %. Furthermore, gender and fitness specific PA intensity thresholds could also be used if HR_max_ is known but VO_2max_ is unknown. In that case, the relationship between HR_max_% and VO_2max_% presented in Fig. 4 could be used to extrapolate VO_2max_% if HR_max_% of an activity is known. Defining intensity of PA in terms of ACSM’s intensity definition expressed as of VO_2max_%, makes it easy for researchers using our thresholds to determine the proportion of the population meeting the current PA recommendation (which are based on intensity).

Unlike other studies, we used only one Actigraph model for derivation of the PA intensity thresholds. Further, our study included elderly adults with a wide range of CRF. This allowed us to better predict the lower and upper bounds for light, moderate and vigorous PA intensity, and to adjust PA intensity thresholds to CRF. In addition we showed, for the first time, that CPM is not only affected by CRF, but also by gender. For this reason we presented different PA intensity thresholds for men and women separately. Importantly, to establish the Actigraph PA thresholds we included flat, uphill and downhill walking. Studies using Actigraph have shown energy expenditure (and thus intensity of movement) to be underestimated during uphill walking and overestimated during downhill walking [30]. Some have suggested that energy over - or underestimation is evened out in terms of overall energy expenditure [30]. However, to represent to the best extent, the real life situation where flat, uphill and downhill walking are all utilized, we included all those states into our main Actigraph PA threshold analysis.

We recognize that our methodology had limitations. We had an uneven subject distribution in the CRF tertiles. However, we accounted for this in the statistical analysis and used weighted averages when creating the relationship between the counts and VO_2max_%. However, once our data was uploaded following VO_2max_ testing, it was realized that too few observations had below 2000 CPM. We, therefore, called some participants back and subjected them to additional measurements (with speeds and inclinations generating CPM lower than 2000) in order to establish the most accurate relationship between CPM and VO_2max_%. Twenty randomly selected participants of the 97 were called back. Eighteen were available and returned back to the lab for additional testing.

Additionally, some of the elderly adults in our study were not very comfortable walking on a treadmill and could have moved unnaturally, potentially producing higher counts. We tried to minimize this by having the study participants familiarize themselves with a treadmill during the warm up period prior to testing. We asked the participants not to hold on to the railing of the treadmill. In case of loss of balance, we stressed that they should gently balance themselves using one hand on the treadmill railing, without exerting too much pressure. It is also important to note that our data were collected during ambulatory activities on a treadmill and may therefore not reflect the activities of free living.

## Conclusions

This study has established new Actigraph thresholds for light, moderate and vigorous PA in terms of VO_2max_% in an elderly population. Importantly, our study takes fitness level and gender into account, and is therefore much more individualized than previously derived PA thresholds for similar populations. We believe that these PA thresholds will be useful in future research on the elderly.

## Abbreviations

ACSM: American college of sports medicine
BMI: body mass index
CPM: counts per minute
CRF: cardiorespiratory fitness
HR: heart rate
MET: metabolic equivalent of task
PA: physical activity
RER: respiratory exchange ratio
VA: vertical axis
VE: ventilation
VM: vector magnitude
VO2max: maximal oxygen uptake
VO2max%: percentage of maximal oxygen uptake
